# Supplementary material for: Association between oral microbiome and seven types of cancers in East Asian population: a two-sample Mendelian randomization analysis
Source: Front Mol Biosci. 2023 Nov 21;10:1327893. doi: 10.3389/fmolb.2023.1327893 (PMC10702768; doi:10.3389/fmolb.2023.1327893)
Supplement: Supplementary file 1 [file DataSheet1.ZIP › Supplementary Materials/MR plots for tongue/tongue═╝/Lung cancer/pheno.14_to_lung cancer_leave_one_out.pdf]

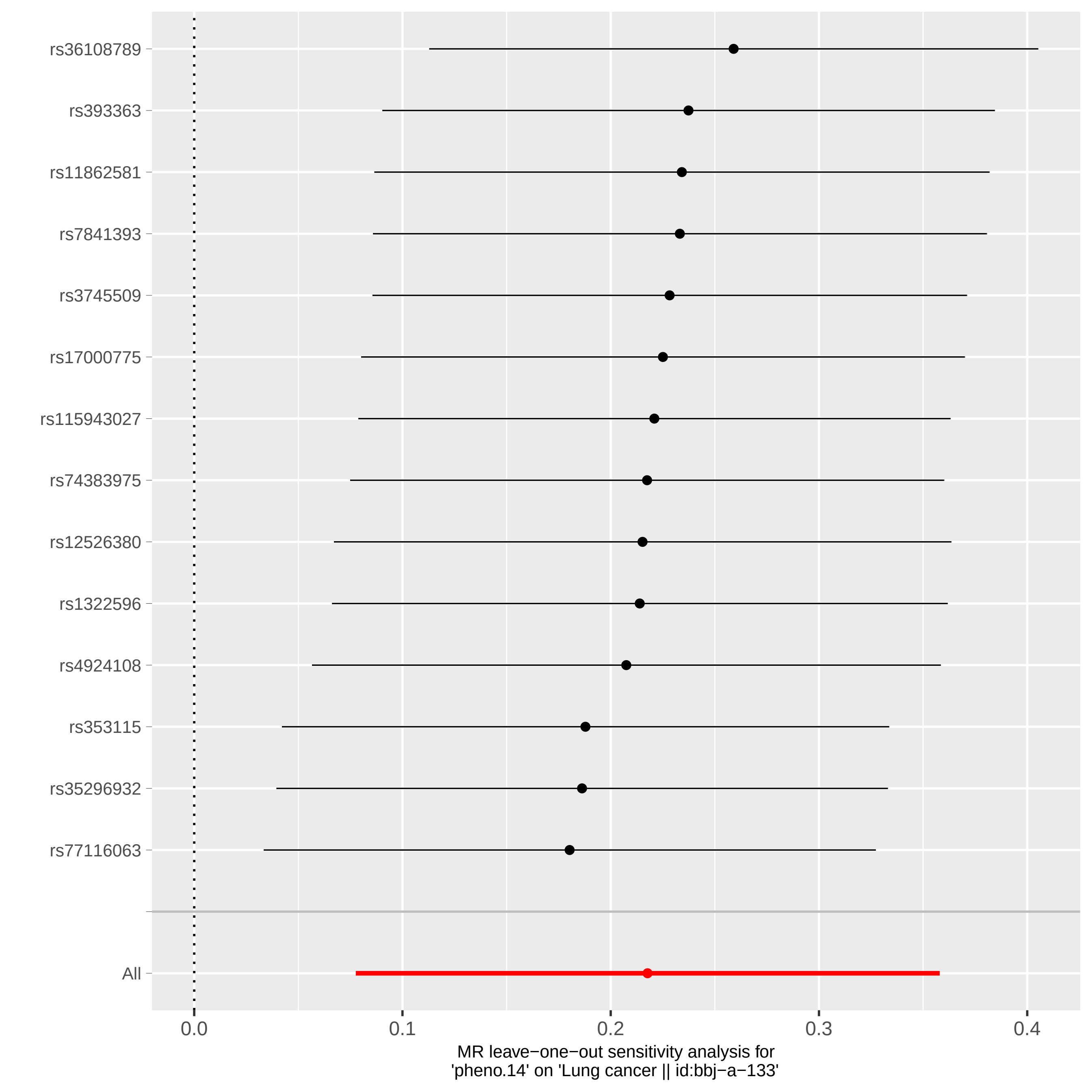
rs36108789
rs393363
rs11862581
rs7841393
rs3745509
rs17000775
rs115943027
rs74383975
rs12526380
rs1322596
rs4924108
rs353115
rs35296932
rs77116063
All
0.0
0.1
0.2
0.3
0.4
MR leave−one−out sensitivity analysis for
'pheno.14' on 'Lung cancer || id:bbj−a−133'
